# Genetics of structural and functional brain changes in autism spectrum disorder

**DOI:** 10.1038/s41398-020-00921-3

**Published:** 2020-07-13

**Authors:** Sheema Hashem, Sabah Nisar, Ajaz A. Bhat, Santosh Kumar Yadav, Muhammad Waqar Azeem, Puneet Bagga, Khalid Fakhro, Ravinder Reddy, Michael P. Frenneaux, Mohammad Haris

**Affiliations:** 1 Functional and Molecular Imaging Laboratory, Sidra Medicine, Doha, Qatar; 2 Department of Psychiatry, Sidra Medicine, Doha, Qatar; 3 Center for Magnetic Resonance and Optical Imaging, Department of Radiology, Perelman School of Medicine, University of Pennsylvania, Philadelphia, PA 19104 USA; 4 Department of Human Genetics, Sidra Medicine, Doha, Qatar; 5 Department of Genetic Medicine, Weill Cornell Medical College, Doha, Qatar; 6 Academic Health System, Hamad Medical Corporation, Doha, Qatar; 7 Laboratory Animal Research Center, Qatar University, Doha, Qatar

**Keywords:** Scientific community, Genomics

## Abstract

Autism spectrum disorder (ASD) is a neurological and developmental disorder characterized by social impairment and restricted interactive and communicative behaviors. It may occur as an isolated disorder or in the context of other neurological, psychiatric, developmental, and genetic disorders. Due to rapid developments in genomics and imaging technologies, imaging genetics studies of ASD have evolved in the last few years. Increased risk for ASD diagnosis is found to be related to many specific single-nucleotide polymorphisms, and the study of genetic mechanisms and noninvasive imaging has opened various approaches that can help diagnose ASD at the nascent level. Identifying risk genes related to structural and functional changes in the brain of ASD patients provide a better understanding of the disease’s neuropsychiatry and can help identify targets for therapeutic intervention that could be useful for the clinical management of ASD patients.

## Introduction

Autism spectrum disorder (ASD) is a neurological and developmental disorder consisting of a wide range of symptoms and disability that develop in early childhood and persists throughout life. The common symptoms of ASD include limited activities, lower engagement and communication, talking and learning problems, and repetitive behavior. According to the World Health Organization, the global burden of ASD is continuously growing, with a current prevalence rate of 1 in 160 children. Reported prevalence rates vary widely from country to country though. Recent data from the Centers for Disease Control and Prevention showed that about 1 in 68 children in the United States had been identified with some form of ASD, with more than 3 million people affected^1^. A recent study estimated prevalence of ASD in the United States in 2014–2016 was 2.47% among adolescents and children^2^. While in the United Kingdom, the annual prevalence rate for children aged 8 years between 2004 and 2010 was 3.8/1000 for boys and 0.8/1000 for girls^3^. Recent studies have shown that the pooled ASD prevalence estimate in Asia is 0.36%, including data from nine countries (China, Korea, India, Bangladesh, Lebanon, Iran, Israel, Nepal and Sri Lanka)^4^. The prevalence of ASD in the Middle East region was documented to be 1.4 per 10,000 children in Oman^5^, 4.3 per 10,000 children in Bahrain^6^, 1/167 in Saudi Arabia^7^, and a recent study reported ASD prevalence to be 1.14 % in children aged 6–11 years in Qatar^8^.

ASD incidence is 4–5 times greater in males than in females^9^. The exact cause of ASD remains unclear; however, it is thought that both genetic and environmental factors play essential roles. The effect of ASD on society is enormous and multifaceted as it affects not only the child but also the siblings and parents and significantly disturbs the functioning of family routine life. Individuals with ASD are very likely to encounter the criminal justice system, mostly due to a lack of knowledge of their social and communication difficulties. There are also financial pressures associated with the recovery and decreased opportunities for jobs. Various studies have focused on the problems faced by families of autistic children^10^. It may affect the performance of the child at school, based on the severity of the disorder, and if untreated or undiagnosed, it may continue into adulthood, affecting both personal and professional life. Although there is no standard treatment for ASD, early detection and use of successful therapies can make a difference in the management and improvement of symptoms, and thus the overall quality of life of children with ASD. Before the clinical symptoms occur, therefore, a consensus is reached in treating ASD based on early detection.

Recent cohort studies have reported that hereditary factors can be traced to more than 50% of autism cases^11^. Historically, the genetic essence of ASD has been identified in family and twin studies^12,13^. The study of family exome sequencing has found some rare de novo mutations in people with ASD as opposed to healthy controls. It is estimated that ~1000 genes are involved in autism^14,15^. On the other hand, the main variants associated with autism^16^ are yet to be identified in genome-wide association studies. By understanding the evidence at gene expression, we will obtain a complete understanding of autism which can aid in the clinical treatment of patients with ASD.

On the other hand, noninvasive imaging provides information on the structural and functional changes of autism in the brain. Studies have shown that connecting genomics to imaging can provide a better understanding of ASD’s pathophysiology^17^ and can help develop therapeutic approaches to prevent or reverse ASD brain changes. In this review article, as demonstrated in the imaging, we illustrate how genetic variations are correlated with the structural and functional changes in the brain.

## Changes in the autistic brain

### Pathological findings

Postmortem studies conducted on autistic brains helped to identify the neuroanatomical changes associated with ASD. Smaller cell size and increased density of cells in the hippocampus, limbic system, entorhinal cortex, and amygdala have been observed in all ages of ASD individuals^18^. Young patients with autism showed abnormally enlarged neurons in the cerebellar nuclei, inferior olive, and vertical limb of the diagonal band of broca^18^. Another postmortem study found an increased number of neurons in the prefrontal cortex in the brain of ASD children^19^. Purkinje cell count was found to be decreased in the archicerebellar and neocerebellar cortices of the cerebellar hemisphere in autistic patients^20^. Another study observed a reduced number of small pale neurons in the brains of adult autistic patients^21^. A postmortem study of the brain of patients with ASD also reported altered axonal density and the presence of impaired myelin in white matter (WM)^22^. Overall, the pathological findings suggest a restricted brain development pattern in ASD patients.

### Structural brain changes

Brain changes in ASD patients have been primarily studied using magnetic resonance imaging (MRI). MRI is the most widely used noninvasive modality to examine the structural and functional brain changes in different neurological and neurodegenerative disorders. Various MR-based methods are applied to study the morphological, functional, and metabolic changes in the brain of ASD. MRI findings in children with ASD ranging from 2 to 5 years of age revealed that there is an abnormal development of frontal and temporal lobes, lower gray matter (GM), WM, and amygdala volume compared to same-age healthy controls^23^. A comparison of the brain morphometric features in 3–4 years old ASD children with typically developing (TD) children and developmentally delayed children^24^ was conducted using high-resolution 3-D MRI. Increased cerebellar volume was found in children with ASD compared to TD, and developmentally delayed children and amygdala enlargement with increased cerebral volume was seen in ASD children^24^. Another study observed cerebral enlargement in both boys and girls with ASD compared to controls, and boys with regressive autism showed more structural alterations as compared to girls^25^. Volumetric MR studies demonstrated a 5–10% increase in brain volume of ASD children who were scanned between 18 months and 4 years^23,26^.

Imaging studies have observed both decreased and increased cortical thickness in patients with ASD. One study, performed on eight autistic subjects, showed decreased cortical thickness in the regions of inferior frontal, occipital, supramarginal, and post- and precentral gyrus, anterior cingulate, prefrontal, parietal, and temporal cortex^27^. Another study showed increased cortical thickness in the parietal and temporal lobes of autistic children compared to healthy controls^28^. An MRI study of adults with ASD, who had delayed language development, also called high‐functioning autism (HFA), observed increased cortical thickness in frontal, occipital, temporal, parietal, cingulate, and fusiform gyri while decreased cortical thickness was observed in the post- and paracentral gyrus^29^. Surface-based morphometry (SBM) analysis is also used to measure cortical thickness in ASD patients. An SBM based study found decreased cortical thickness in the left orbitofrontal and parahippocampal gyrus, left frontal pole, and pars triangularis, while increased cortical thickness was observed in the left precuneus and anterior cingulate cortex^30^. Another SBM based study reported reduced cortical volume in the left middle temporal gyrus, reduced gyrification index in the left supramarginal gyrus and increased cortical thickness in pars opercularis of inferior frontal gyrus in HFA compared to TD individuals^31^. The SBM approach was also used to compare the cortical abnormalities in individuals with low‐functioning autism (LFA) and HFA and Asperger’s syndrome with TD controls. In HFA individuals, shape abnormality was observed bilaterally in parietal operculum and ventral postcentral gyrus. LFA individuals showed shape abnormality in the pars opercularis located in the inferior frontal gyrus, and individuals with Asperger’s syndrome showed bilateral abnormalities in the intraparietal sulcus region^32^. Voxel-based morphometry analysis observed increased GM volume in frontal, parietal, temporal lobes and limbic system and reduced WM volume in limbic system, frontal and temporal lobes of autistic subjects^33^. Another voxel-based morphometry study showed reduced GM volumes in frontal, temporal, and parietal lobes in HFA individuals compared to healthy controls^34^.

Diffusion tensor imaging (DTI), a noninvasive MRI technique, exploits the diffusion properties of a water molecule to map the microstructural, architectural, and compositional characteristics of the tissue. Fractional anisotropy (FA) and mean diffusivity are two commonly used DTI metrices in mapping the restricted movement of water molecules in the tissue, where higher FA associates with the greater directionality and tissue integrity^35^. DTI is widely used to track the changes in the microstructural properties of WM such as axonal density, axonal injury, and myelination in various human brain’ disorders. Provided that ASD is a developmental disorder, DTI can be used to identify variations in WM to better define developmental trajectories in individuals with ASD^36^. Increased FA was observed in the frontal lobes and corpus callosum regions of children aged 2–3 years with ASD compared to healthy controls^37^. While, another study showed reduced FA in frontal–posterior tracts in the brain of children with ASD, aged 10–18 years^38^. DTI studies showed a difference in trajectories of FA in infants aged 6–24 months who were at higher family risk of developing ASD. FA was higher in infants who had ASD diagnosis at the age of 6 months, while FA was found to be lower at the age of 24 months^39^. One study reported decreased FA in the parietal lobes, temporal lobes, lateral occipital cortex, left anterior cingulate, middle frontal cortex, and in the corticospinal tract of individuals with HFA as compared to the controls^34^. DTI tractography confirmed abnormality of the WM structures in the regions of the corpus callosum, inferior longitudinal and fronto-occipital fasciculus, and superior longitudinal fasciculus in ASD patients^40^.

## Genes affecting brain morphology

### Preclinical studies

While there may be several specific genes affected in autism, there is increasing evidence that these genes converge on a limited number of biological pathways that include changes in cortical development, synapse function, transcription and translation, chromatin modification, and microglial activation^41,42^ (Table 1).

**Table 1:** ASD-associated genes affecting brain morphology and function.

Gene (s)	Risk allele/polymorphism	Subjects	Method used	Brain region affected/potentially could be affected	Findings
*ANK2*		*ankB* mutant mice	DTI	Cortex	Increased interhemispheric asymmetry in the cortex and increased overall connectivity^46^
*HOXA1*	*HOXA1 A218G* polymorphism	Autistic individuals and ethnically matched controls	Family-based association analyses	Could potentially affect hindbrain neural networks that could, in turn, affect brainstem	Enlarged cranial circumference^68,143^
*HOXA1 HOXB1*		Mice mutants of *HOXA1/HOXB1*	Immunohistochemistry, RNA in situ hybridization, SEM, cell apoptotic and proliferation assay	Hindbrain	Loss of rhombomere 4 and 5 and loss of second branchial arch in *HOXA1/HOXB1* mutant mice^53^
*PTEN*		ASD and macrocephaly	Gene mutation analysis	Cerebral cortex (as one of the subject’s brain MRI showed dilatation of perivascular spaces in the basal ganglia)	Larger head circumference^72^
*CHD8*		ASD and Developmental delay	Analysis of *CHD8* gene expression using Zebrafish model	Forebrain and midbrain	Increased head size^55^
*CNTNAP2*	*rs779475*	Healthy controls	DTI, sMRI	Frontal lobe, occipital lobe, cerebellum	Reduction in WM and GM volumes in the cerebellum, frontal and occipital lobes in homozygotes for the risk allele^77^
	*rs2710102*	ASD and TD individuals	fMRI (reward-guided implicit learning task)	Frontal cortex	Reduced mPFC activation in nonrisk individuals and increased frontal connectivity in the risk allele carriers^117^
	*rs7794745 T rs2710102 C*	Healthy individuals	fMRI (language task)	Prefrontal cortex, temporal cortex	Increased activation of the right inferior frontal gyrus and right lateral temporal cortex in the risk allele carriers^118^
	*c.3709DelG mutation*	Individuals with syndromic ASD and healthy controls	MRI	Prefrontal cortex	Increased head circumference and GM volume^76^
			Forebrain organoid culture		Increased total brain volume in individuals carrying the *CNTNAP2 c.3709DelG* mutation^76^
		*CNTNAP2* knockout mice	Laser-scanning photostimulation, whole-cell recordings, and electron microscopy	Prefrontal cortex	Reduced excitatory and inhibitory synaptic inputs onto L2/3 pyramidal neurons in the mPFC of *CNTNAP2* knockout mice with reduced spines and synapses^144^
*MET*	*rs1858830*	Healthy individuals	sMRI	Frontal lobe, temporal lobe, anterior cingulate cortex	Reduction in cortical thickness with increasing C allele dose in temporal gyri, ventral pre- and postcentral gyri, anterior cingulate and in fronto-polar cortex regions^78^
	*rs1858830*	ASD and TD individuals	fMRI (observation of emotional faces), resting-state fMRI, DTI	Neocortex	Higher activation of amygdala and striatum and reduced WM integrity and intrinsic connectivity between the posterior cingulate cortex and mPFC regions in MET risk allele carriers^79^
*SLC6A4*	*5HTTLPR*	ASD children	MRI	Cerebrum	*5HTTLPR* short allele associated with increasing cerebral GM volumes^80^
*NRXN1*	*rs1045881*	ASD and schizophrenia risk allele carriers	sMRI	Frontal lobe	Reduction in frontal WM volumes and altered sensorimotor function^115^
	*bi-allelic NRXN1-α del*	ASD individual and healthy controls	Single-cell RNA sequencing		Reduced proliferation capability and calcium signaling and high expression of radial glia-like morphology in *NRXN1-α* del NES cells^116^
*OXTR*	*rs2254298A*	Healthy Japanese adults	MRI	Amygdala	Larger bilateral amygdala volume^81^
	*rs2254298(G*>*A)*	Healthy females	MRI	Amygdala, anterior cingulate cortex, brainstem	Increased amygdala and GM volume in the brainstem and decreased total GM volume and GM volume in the anterior cingulate cortex in G/A heterozygotes^82^
	*rs53576*	Healthy adults	sMRI and fMRI	Hypothalamus and amygdala	Decrease in hypothalamus GM and amygdala activation and increased functional correlation of hypothalamus and amygdala in minor allele carriers for *rs53576*^83^
	*rs2254298A*	Healthy individuals	MRI	Cerebral cortex	Reduced GM volume in the right insula in males with the risk allele^84^
	*rs2268498 T/C*	Healthy individuals	fMRI (fear processing task)	Occipital lobe	T-allele homozygotes showed increased activation of inferior occipital gyrus during recognition of fear expressions^86^
	*rs2268493*	Healthy individuals	fMRI (reward anticipation task)	Nucleus accumbens, amygdala, insula, thalamus, and prefrontal cortical regions	T-allele homozygotes showed reduced activation in the mesolimbic reward circuitry^85^
	*rs401015*	Healthy individuals	fMRI (direct gaze processing task)	Amygdala	Heterozygotes CT variants showed increased amygdala activity^87^
	*rs2254298A*	Healthy individuals	VBM, fMRI	Hypothalamus, dorsal anterior cingulate cortex	Decreased hypothalamus GM volume and deactivation of dorsal anterior cingulate gyrus and increased structural coupling of dorsal anterior cingulate gyrus and hypothalamus in A carriers^88^
	*rs53576*	Healthy individuals	VBM, fMRI (mentalizing paradigm)	Amygdala, parietal lobe	Higher brain GM volume in the left amygdala and lower GM volume in superior parietal lobule^89^
*CDH9 and CDH11*		Mice	In situ hybridization	Cerebellum	High expression of *Cdh11* in the central part and *Cdh9* in the surrounding areas in lobules VI/VII and Crus I and Crus II regions of the cerebellum^145^
*RELN*		Autistic and healthy individuals (postmortem samples)	SDS-PAGE, Western blotting	Cerebellum	Significant reduction of Reelin protein in the cerebellar region of ASD individuals^132^
*SLC25A12*		Autistic and healthy individuals (postmortem samples)	qRT-PCR, RNA extraction, in situ hybridization	Prefrontal cortex	Strong expression of *SLC25A12* in the BA46 prefrontal cortex of autistic subjects^146^
*PRKCB1*	*rs3785392* *rs3785387*	Autistic and healthy individuals (postmortem samples)	qRT-PCR, Western blotting, Microarray analysis	Cerebral cortex	Significant reduction of *PRKCB1* gene expression in the temporal neocortex of autistic individuals^147^
*TAOK2*		Mice	MRI, DTI	Midbrain, thalamus, hypothalamus and hindbrain regions	Enlarged volumes of the midbrain, hindbrain, hypothalamus, thalamus, cerebellum, and hippocampus and reduced density of fiber tracks in the medial corpus callosum of *TAOK2* knockout mice^57^
*MECP2*		Autistic and healthy individuals (postmortem samples)	Immunofluorescence and laser-scanning cytometry	Frontal cortex, temporal and occipital lobe	Decreased *MECP2* expression in the frontal cortex and fusiform gyrus of autistic individuals^148^
*SHANK3*		*SHANK3* transgenic Mice	MRI	Hippocampus, forebrain, and midbrain	Reduction in total brain volume and hippocampal size and increase of basal ganglia in *SHANK3* knockout mice as compared to prenatal zinc-deficient mouse model of ASD^45^
*NL3*		*NL3* knockin mice	Ex vivo MRI and DTI	Caudate putamen, substantia nigra, somatosensory cortex, corpus callosum, internal capsule, and cerebral peduncles	Decreased volumes of WM and GM regions in the *NL3* knockin mice as compared to wildtype mice^43^
*AVPR1A*	*RS1, RS3*	Healthy individuals	fMRI (face-matching task)	Amygdala	*RS3 334bp, RS3* longer variants, *RS1 312* bp, and *RS1* shorter variants risk allele carriers showed stronger activation of left amygdala^120^
*MECP2* *ITGB3* *NL3*		*MECP2 308* truncation, *ITGB3* knockout, *NL3* knockin mouse models	MRI	Cerebellum	Increased GM and WM volume in crus II lobule and GM volume in paraflocculus in *NL3* knockin mice, Expansion of vermis lobules III–X, the anterior lobule, the paraflocculus, and simple, crus I, and crus II hemisphere lobules in the homozygous *MECP2* model and reduced cerebellum volume in *ITGB3* knockin mice^44^
*CD38*	*rs3796863*	Healthy individuals	fMRI (social, emotional stimuli and gaze processing task)	Temporal cortex	Higher activation of the left fusiform gyrus in homozygous risk allele carriers^119^
	*rs3796863*	Healthy individuals	fMRI (social cognition tasks)	Amygdala	Epistasis effect between CD38 and COMT in the amygdala^121^
		C57BL/6 mice and CD38^*−/−*^ mice	MRI	Prefrontal cortex	Larger whole brain volume, abnormal cortex development, and impaired synaptic plasticity in the prefrontal cortex of CD38^*−/−*^ mice^108^

*MRI* magnetic resonance imaging, *DTI* diffusion tensor imaging, *fMRI* functional magnetic resonance imaging, *VBM* voxel-based morphometry.

Neuroligin-3 (*NL3*) is a major cell adhesion protein that plays a vital role in the development of the synapse, and it has also been implicated in ASD. An ex vivo MRI study showed a reduction in various WM and GM regions of *NL3* knockin mice, with deficits in social and anxiety-related behaviors^43^. A similar study used MRI to image mouse models with mutations implicated in autism, such as *NL3* knockin, *MECP2*, and integrin β3 (*ITGB3*) homozygous knockout mouse models to investigate the effects of these mutations on brain morphology^44^. The study found enlarged WM, and GM volumes of crus II lobule in *NL3* mutants increased cerebellar volumes in *MECP2* mutants and reduced cerebellar structures in *ITGB3* mutants^44^. A neuroimaging study reported reduced total brain volume and hippocampal size with enlarged basal ganglia structures in *SHANK3* knockout mice compared to a prenatal zinc-deficient mouse model of ASD^45^.

A recent study on mice with *ANK2* mutations showed an increased number of excitatory synapses during postnatal development with increased axonal branching, which supports the presence of altered connectivity and penetrant behavioral impairments in mice and humans carrying ASD-related *ANK2* mutations^46^. Mutations in *CHD2* have been linked with various neurodevelopmental disorders, including ASD^47^. *CHD2* is shown to play an essential role in brain development as the suppression of *CHD2* can inhibit the self-renewing ability of radial glial cells and can increase the generation of intermediate progenitor cells and neurons during the process of neurogenesis that might contribute to abnormal neurodevelopment^48^. Another study showed that *CHD2* knockdown zebrafish exhibited altered locomotor activity, which contributed to epileptic encephalopathy^49^. These studies show that there is a genetic overlap found between ASD disorder and other neurological disorders such as schizophrenia, epilepsy, and ID^50^. In addition, knockdown of *CHD8* resulted in reduced axon and dendritic growth with delayed neuronal migration in mice^51^. *CHD8* suppression in zebrafish affected the gene expression in neurodevelopmental pathways related to the proliferation of neural progenitor cells^52^.

One of the studies showed that mice mutants of *HOXA1/HOXB1* had several defects, including the loss of rhombomere 4 and 5 and the loss of 2nd branchial arch affecting the development of the hindbrain^53^. Previous studies have shown the role of *16p11* deletions^54^ and the *CHD8* gene^55^ in the brain overgrowth in idiopathic ASD. The postnatal development mechanisms such as pruning and dendritic arborization are affected by the overproliferation of cortical progenitor cells. The overproduction of neocortical neurons also contributed to the autistic-like features in mice^56^. Studies have reported that *16p11* deletions resulted in the alteration of cortical progenitor cell proliferation in mice and increased brain volumes in individuals with syndromic ASD^54^. One of the genes associated with the *16p11.2* region known as *TAOK2* has been found to cause ASD-related cognitive abnormalities^57^. A neuroimaging study found dosage-dependent abnormalities in *TAOK2* heterozygous and knockout mice. MRI of the *TAOK2* knockout mice revealed enlarged midbrain, hindbrain, hypothalamus, thalamus, cerebellum, and hippocampus volumes, with a regional delay in the development of the neuronal track in the medial corpus callosum^57^.

Animal studies have shown that the MET protein regulates early cortical development in the neurobiology of ASD. A study by Qiu et al. showed excitatory hyperconnectivity in neocortical circuits of *Met* conditional knockout mice^58^. While another study showed that the reduction of MET and HGF/SF correlated with calbindin+ interneurons in the cortex of urokinase-type plasminogen activator receptor knockout mouse, thereby affecting cortical development^59^.

### Clinical studies

In the clinical setting, several imaging studies were conducted that link specific gene polymorphisms with abnormal brain morphology in ASD patients (Table 1). Gene deletions such as *22q11.2* and *16p11.2* are associated with ASD. These deletions are also found to be linked with other neuropsychiatric disorders, including DiGeorge Syndrome, conotruncal anomaly face syndrome, velocardiofacial syndrome, intellectual disability, and schizophrenia^60,61^. *22q11.2* deletion in patients having high-risk negative symptoms at risk of developing schizophrenia showed decreased gyrification mainly in the medial occipital and temporal regions, involved in social cognition and early visual processing^62^. Another study reported increased cortical thickness in the frontal lobe, cerebral cortex, and superior parietal lobes and decreased cortical thickness in the regions of superior temporal gyrus and posterior cingulate cortex in patients with *22q11.2* deletion syndrome^63^.

**Fig. 1:**
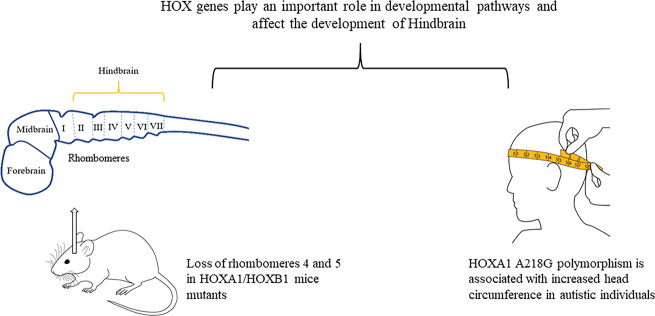
Structural brain changes associated with *HOX* genes. *HOXA1* A218 gene polymorphism is associated with an increased head circumference in autistic individuals, and *HOXA1/HOXB1* mice mutants showed loss of rhombomeres 4 and 5 affecting the hindbrain development^53,68^.

Head circumference is abnormally increased in ASD patients^64^. Many factors can be responsible for the brain overgrowth in ASD, such as altered synapses during the development process, increased neurites or the number of neurons in the brain. *HOX* genes play an essential role in the development of organs and are responsible for the proper positioning of organism’s segment structures during embryonic development (Fig. 1). Out of all the *HOX* genes, *HOXA1* is a critical gene that is involved in the development of the central nervous system, internal ear, and hyoid bones^65,66^. *HOXA1* polymorphism has been found to induce an increased head growth rate in autistic and TD children^67^. In addition, autistic individuals with *HOXA1 A218G* polymorphism displayed an enlarged cranial circumference^68^, while in autistic patients, one study reported minor contributions of the *HOXB1* gene to head circumference^69^.

Mutations in *PTEN* have also been associated with brain morphological changes in ASD. *PTEN* mutations have been observed in Cowden syndrome, and macrocephaly is the main feature of patients with Cowden syndrome, and some of the individuals with this syndrome are also found to be autistic^70,71^. Sequencing of the *PTEN* gene in ASD patients revealed three heterozygous germline mutations^72^ and a missense mutation^73^. An ASD-related syndrome, known as cortical dysplasia-focal epilepsy (CDFE) is a rare neuronal migration disorder, and patients with CDFE exhibit autistic characteristics. A study reported a homozygous mutation in the *CNTNAP2* gene in all CDFE patients, which is responsible for an early developmental insult, mainly in the frontal and temporal neocortex^74^. Another study showed that the impact of *CNTNAP2* missense variants on axonal growth as the loss of *CNTNAP2* allele contributes to the disruption in axonal growth^75^. An MRI study investigated the homozygous *c.3709DelG* mutation in *CNTNAP2* in the forebrain organoids generated from human induced pluripotent stem cells that were derived from patients with syndromic ASD. The study found that individuals with *c.3709DelG* mutation in *CNTNAP2* displayed increased head circumference and increased GM volume^76^. An imaging genetic study found an association of *CNTNAP2 rs779475* single-nucleotide polymorphism with WM and GM morphology in healthy controls and observed reduced volume of GM in the regions of frontal and occipital lobes and cerebellum. However, there was a reduction in the WM volume in the regions of right rostral cingulum, right caudal inferior fronto-occipital fasciculus, and the posterior thalamic radiations in homozygotes for the risk allele (*rs779475 T/T*) as compared to the nonrisk homozygotes and a group of *rs779475T* heterozygotes^77^.

Mutations in the chromodomain helicase DNA binding protein 8 (*CHD8*) gene, which plays a vital role in the chromatin remodeling process, are amongst the most commonly reported mutations in ASD. *CHD8* mutations have also been linked with axonal and dendritic growth, and a study showed that *CHD8* is highly expressed in a microtubule-associated-protein 2 positive neurons and parvalbumin neurons which plays an essential role in the development of neocortex in the human brain. The findings of the study suggested that *CHD8* mutations in humans can result in neuronal deficits that can contribute to ASD pathophysiology^51^.

Studies have proposed the association of MET proto-oncogene, receptor tyrosine kinase (*MET*), as a candidate risk gene for ASD. In order to find the association of MET with cortical development, a structural MRI study investigated the relationship between a single-nucleotide polymorphism within the MET promoter (*rs1858830, G*→*C*) and the development of cortical thickness in ASD individuals. The study observed cortical thickness reduction in the regions of ventral precentral and postcentral gyri, superior and middle temporal gyri, anterior cingulate cortex, and in the right fronto-polar cortex with increasing C allele dose^78^. Another study found that *MET* risk allele carrier (*rs1858830*) individuals with ASD and TD showed higher activation of amygdala, reduced WM integrity, and reduced connectivity between the posterior cingulate cortex and medial prefrontal cortex (mPFC)^79^.

A common polymorphism in the serotonin-transporter gene (*SLC6A4*) involving a serotonin-transporter related polymorphic region (5HTTLPR) results in the generation of short or long alleles. A study by Wassink et al. found that the 5HTTLPR short allele significantly influences the cortical GM volumes in male autistic children^80^. Several neuroimaging studies have investigated the effects of different oxytocin receptor (*OXTR*) risk alleles (*rs2254298, rs53576, rs226849, rs401015*) on brain morphological changes in healthy adults^81–89^. *OXTR* risk allele carriers (*rs2254298*) showed increased amygdala volume^81,82,89^, decreased GM volume in the hypothalamus^83^, and right insula^84^. A study showed that dysregulation in the β-catenin/BRN2 transcriptional cascade was associated with an increased number of neurons and proliferation of neural progenitor cells derived from pluripotent stem cells of ASD individuals and also contributed to increased brain volumes^90^.

### Functional brain changes

Resting-state fMRI (rsfMRI) is a powerful tool that is being used for mapping the neuronal activity of various neurological disorders. rsfMRI studies have revealed abnormalities in the neuronal network in ASD patients that help in understanding the underlying neurobiology of ASD. An rsfMRI study observed altered functional connectivity between the cerebral cortex and dentate nucleus in individuals with ASD, which is suggestive of impaired interaction between the cerebellum and social cortical regions of the brain^91^. Another study observed reduced integration of the default mode network with the mPFC and angular gyrus regions in the ASD group^92^. The mPFC region is mainly involved in decision making, and one of its primary function is encoding task-related information in the working memory^93^, while angular gyrus is involved in semantic processing, default mode network, social cognition, and memory retrieval and attention^94^. Another rsfMRI study showed that the underconnectivity in the temporal sulcus, which is a human voice processing region, in ASD children in addition to a decreased connectivity in reward-related regions in the brain of ASD children. While task-based fMRI showed altered network connectivity in multiple brain regions that control social and emotional processing in ASD^95^. A sentence comprehension task-based study showed decreased connectivity in cortical areas of ASD individuals, which signifies the low congruence of brain regions that are essential for integrating information^96^. Other task-based studies have also reported decreased connectivity in the fronto-parietal regions that are involved in working memory (n-back working memory task)^97^, facial processing (emotional facial processing task)^98^, response inhibition (simple inhibition/1-back inhibition task)^99^ and visuomotor coordination (measuring BOLD signal)^100^ in ASD individuals. Task-based study based on social processing found hypoactivation in social brain nodes in the regions of the mPFC, frontal gyrus, insula, temporal sulcus, amygdala, and the fusiform gyrus^101^.

Task-based fMRI studies focused on cognitive control reported fronto-striatal dysfunction in ASD, specifically in frontal gyrus, basal ganglia, and anterior cingulate cortex regions. Task-based studies that involved examining brain responses associated with figures of speech like puns^102^ and examining the neural activity during the detection of senseless sentences^103^ have also shown communication impairments such as decreased brain synchrony in language processing regions in ASD^102,103^. Task-based studies focusing on reward processing showed impairments in mesolimbic and meso cortical brain regions in ASD. Both over connectivity and underconnectivity between frontal and posterior regions was observed in task-based functional connectivity study^104^. An fMRI study revealed that ASD individuals had reduced activation of higher-order social (fusiform, cingulate, amygdala, gyrus, insula) and executive processing (prefrontal cortex) of the brain and higher activation of lower-order structures that are involved in the mediation of primary motor and sensory processing during emotional task performance^105^. While some task-based studies such as source recognition task^106^ and emotional face processing task^107^ have reported increased connectivity between the brain regions of ASD individuals^106,107^.

## Limitations of functional imaging in ASD

Neuroimaging can help bridge the gap between genes, environment, and different ASD behavioral phenotypes. Although functional imaging techniques such as fMRI have revolutionized the understanding of various mental disorders, the fMRI technique also contains several limitations. The big challenge in current fMRI-based methods is the impact of age/gender and the development of scanning techniques for the diagnosis of ASD across heterogeneous populations. One of the limitations of fMRI is that it has no role in measuring the direct neuronal activity instead it provides changes in the signal based on the blood oxygen level, which may lead to the false-positive or -negative signal specially in patients with abrupt behavioral changes such as ASD. Secondly, an individual’s performance on a specific task must be considered in a task-based fMRI and must be well-thought-out when analyzing the fMRI data. The fMRI data are also analyzed as group averages; therefore, it is not straightforward to diagnose individual autistic patients. This could be par by introducing an individualized diagnostic model based on artificial intelligence.

Furthermore, the data obtained from fMRI may be compromised to some extent due to head motion artifacts that alter voxel and stable state magnetization leading to distortion of anatomical locations and may show false regions associated with brain activity. In addition, fMRI data can only demonstrate that a particular region of the brain is involved in a specific cognitive function but cannot determine whether it is a cause or a consequence of that specific cognitive function. The lack of longitudinal data also makes it challenging to identify the abnormal developmental course in ASD during infancy. Nevertheless, the integration of multiple sites and data sources and the increase in the number of subjects and intra-variability among subjects can improve the feasibility of the fMRI technique in ASD.

## Genes affecting brain functions

### Preclinical studies

Reduced levels of oxytocin have been found in ASD patients, and CD38 is a vital enzyme that controls oxytocin production. A CD38^−/−^ mice study showed abnormal development of the cortex and impaired synaptic plasticity in the prefrontal cortex region with impaired social and emotional responses^108^ (Table 1). Genetic variations in the 1A gene of the arginine vasopressin receptor (*AVPR1A*) were linked to autism. Impaired social recognition and reduced anxiety behaviors were observed in knockout mice of *AVPR1A*, while overexpression of *AVPR1A* in mice led to increased social memory^109,110^. There is also an inversion of cortical layering due to neuronal migration defects in the Reelin (*RELN*) knockout mice^111^, resulting in various behavioral abnormalities, such as aggressive behavior and memory and learning impairments^112^. CNVs of the cytoplasmic FMR1-interacting protein 1 (*CYFIP1*) was found to be associated with ASD and other neuropsychiatric disorders such as schizophrenia. CYFIP1 plays an essential part in regional brain connectivity and corpus callosum function. A study found that the *CYFIP1*-heterozygous mice displayed reduced bilateral functional connectivity across the entire brain and defects in the WM architecture. In these mice, reduced myelination is also observed in the callosal axons, an altered presynaptic feature that contributes to the motor and sensorimotor abnormalities^113^.

Another rsfMRI study showed decreased local and long-range functional connectivity in prefrontal and midbrain of mice with the homozygous loss of *CNTNAP2* and may contribute to the development of autistic-like features and other neurodevelopment disorders^114^.

### Clinical studies

**Fig. 2:**
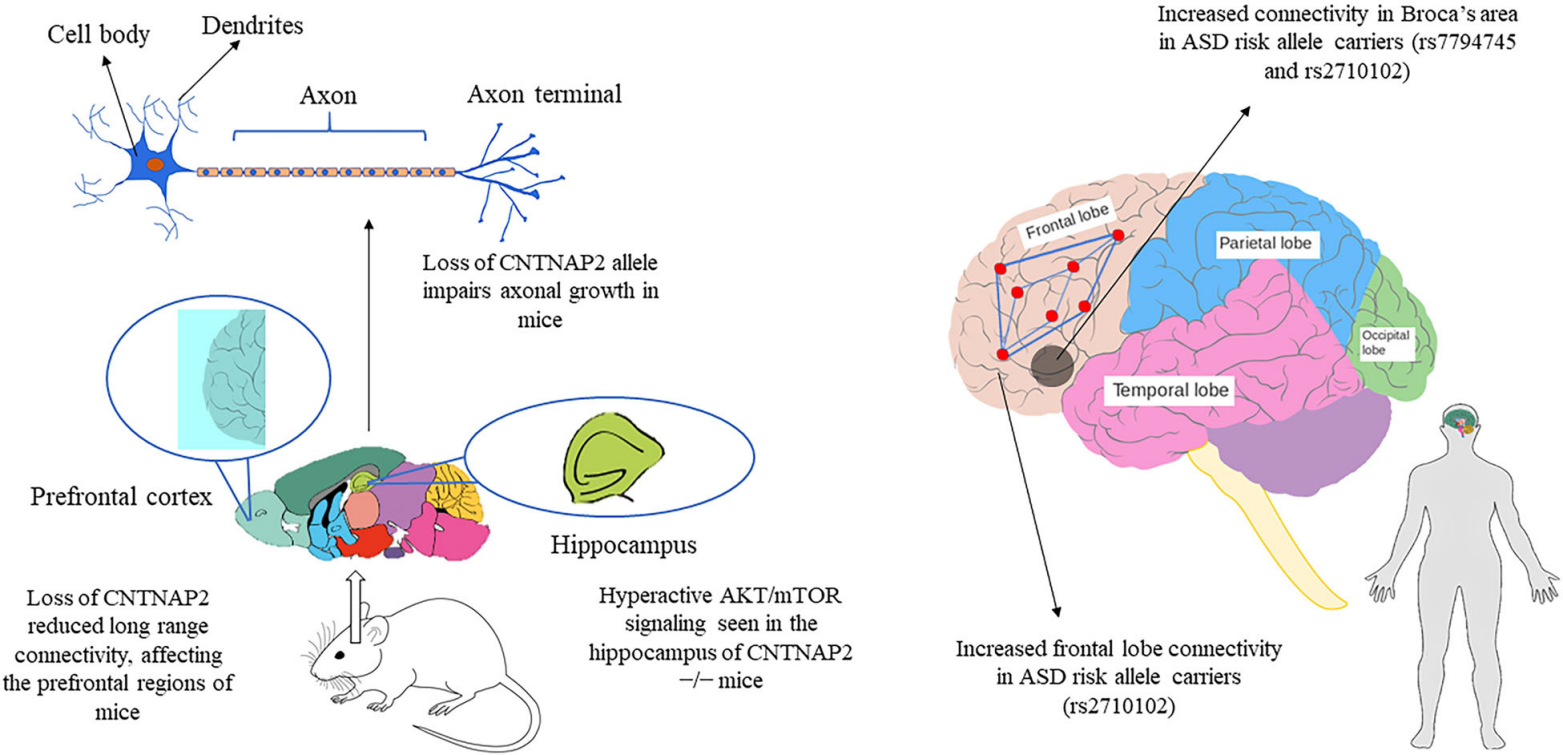
Specific studies in humans and mice with ASD showing functional brain changes correlated with *CNTNAP2* gene. The *CNTNAP2* gene is associated with increased frontal lobe connectivity^117^ and increased connectivity in ASD risk allele carriers within Broca’s area^118^. Studies on mice show that the loss of *CNTNAP2* gene impairs axonal growth^75^, reduces functional connectivity in the prefrontal cortex^114^, and hyperactive AKT/mTOR pathway^149^.

**Fig. 3:**
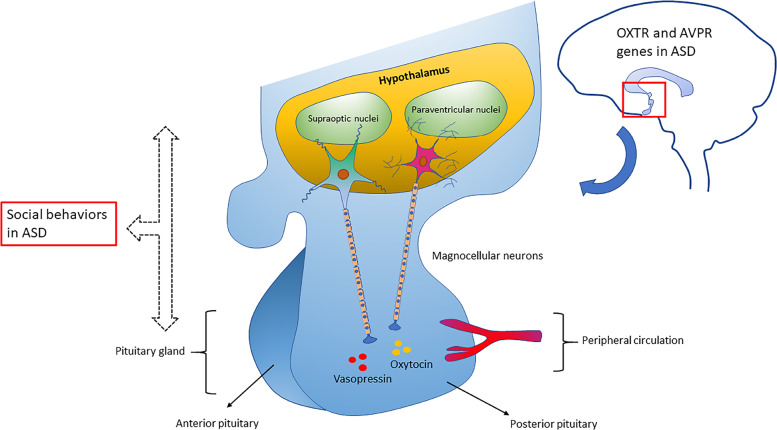
Oxytocin and vasopressin (OT-AVP) pathway associated with social behavior in ASD. In the hypothalamus, magnocellular neurons originating from paraventricular and supraoptic nuclei release oxytocin and vasopressin in the posterior pituitary which then goes into the peripheral circulation. Any deficiencies in oxytocin or vasopressin levels contribute to ASD’s social behavioral impairments.

Imaging genetics studies have shown the involvement of ASD risk genes in altering the brain circuits that control reward and language processing, and social behavior (Table 1). Neurexin-1 (*NRXN1)* is one of the ASD risk genes that are found to influence the brain structure and functions, and its polymorphisms are found to be associated with structural alterations in the prefrontal–thalamic circuitry in healthy individuals, conferring a potential risk of developing ASD or schizophrenia^115^. Single-cell analysis of neural stem cells from an autistic patient with bi-allelic *NRXN1*-α deletion observed a phenotypic shift toward radial glia-like cell with impaired maturation action potential and reduced calcium signaling^116^. On the other hand, *CNTNAP2* polymorphisms are found to be associated with altered brain connectivity in regions that are involved in reward and language development^117^ (Fig. 2). Healthy individuals carrying the risk alleles for ASD and language impairment (*rs7794745 T, rs2710102 C*) showed increased activation in the right inferior frontal gyrus and right lateral temporal cortex^118^. Mutations in the genes involved in the oxytocin/vasopressin system may also contribute to ASD risk as they influence the function of brain regions such as the amygdala and hypothalamus that are associated with emotional and social processing^119,120^ (Fig. 3). Studies have found an association between a common genetic variant in *CD38* (*rs3796863*), autism, and the impact of oxytocin levels on brain response to social stimuli^119,121^. An fMRI study observed increased activation of the left fusiform gyrus in homozygotes for *CD38* genetic variant *rs3796863*^119^. The *MET* gene variants are also found to be involved in altering the connectivity and integrity of WM in the temporo-parieto-occipital regions that are involved in high neurological functions such as working memory, language processing, and face and object recognition, all that may predispose an individual to ASD^79^. Another study reported that the loss of *CNTNAP2* contributed to the reduction in long-range connectivity in the brain regions of mice, mainly affecting the prefrontal regions that act as connectivity hubs in the brain^122^ and this hypoconnectivity in the fronto-posterior region lead to impaired social behaviors, predisposing to autism risk^114^. The *OXTR* polymorphism *rs1042778* is found to be associated with two common clinical phenotypes of ASD, such as aggression and panic in male ASD patients. As compared to *GG* homozygotes, the *T*-allele carriers were at higher risk of possessing aggressive and panic behaviors^123^. Some of the task-based fMRI studies in healthy individuals carrying the *OXTR* risk alleles showed increased activation of inferior occipital gyrus during a fear processing task^86^, decreased activation in the mesolimbic reward circuitry in a reward anticipation task^85^ and increased amygdala activity during a direct gaze processing task^87^. One study found elevated levels of AVP with reduced levels of apelin in ASD patients that point to the vasopressinergic dysfunction in autism^124^. Another study proposed that the shorter alleles of *RS1* polymorphism lead to reduced transcription of *AVPR1A* that may increase the susceptibility to ASD^125^. An fMRI study found an association between ASD and *AVPR1A* genetic variants and reported differential activation of amygdala during a face-matching task in *RS1* and *RS3* risk allele carriers^120^.

Reduced expression of three genes namely metaxin 2 (*MTX2*), neurofilament light polypeptide (*NEFL*), and solute carrier family 25, member 27 (*SLC25A27*) was found in the anterior cingulate gyrus, the motor cortex, and thalamus regions of autistic patients. *NEFL* plays a vital role in the maintenance and assembly of the axonal cytoskeleton^126^, while *SLC25A27* is expressed in the central nervous system and has been found to play an essential role in neuronal cell differentiation^127^, apoptosis inhibition^128^, and reduction of reactive oxygen species^129^.

One of the critical regulators of neuronal migration is RELN glycoprotein, shown to play a vital role in the neuron layering. Aberrant reelin signaling has been reported in many studies in individuals with ASD^130–132^. *RELN* mutations disrupt neuronal migration and connectivity and cerebellar hypoplasia^133^. A significant reduction in the RELN protein has also been observed in the cerebellum of autistic individuals^132^. Another gene *T-Box Brain Protein 1* that encodes a brain-specific T-box transcription factor plays an essential role in the neuronal development, axonal migration, and the development of the cerebral cortex and amygdala^134^. This gene has been identified as an ASD causative gene^135^ and is involved in the differentiation of neurons during the early development of neocortex^136^.

## Diagnostic models of ASD

### Diagnostic models based on MR-derived features

MRI-based diagnostic models are used for the behavioral assessment of autistic patients. These diagnostic model studies involve three steps, including extraction of properties from MR images, construction of diagnostic model using statistical models followed by evaluation and validation by researchers. Several studies have focussed on MRI-based diagnostic models for the detection and classification of ASD^30,137^. Diagnostic model performance is strongly influenced by types of entities selected as components of the model. For example, a study showed the comparison of diagnostic models based on regional thickness derived from surface morphometry (SBM) with diagnostic models based on volumetric morphometry involving four different classification methods and classification based on thickness was found to be more efficient and predictive of ASD compared to classification based on volume^30^. rsfMRI pipelines have been used to extract predictive biomarkers in autism by constructing participant-specific connectomes and then comparing these connectomes across participants to learn connectivity patterns that may identify ASD individuals^138^. The results suggested that rsfMRI data collected from different sites could reveal robust functional connectivity biomarkers of ASD.

### Diagnostic models based on imaging genetics

Imaging genetics in ASD has proven useful, and pathways that include common genetic variation in TD individuals at risk of developing ASD have been characterized. Prenatal transcription regulation and synapse formation in the developing brain is impacted by the genes associated with ASD (Table 1). Alteration in frontal WM connectivity and structure and disturbance in the frontal, temporal, and occipital circuits involved in visual and language processing was found to be associated with *NRXN* superfamily genes^115^. Neuropeptide signaling and emotional functioning was found to be influenced by the oxytocin and arginine vasopressin receptor genes via structural and functional modification in the amygdala–hypothalamus circuitry^77^. One study showed a relationship between frontal lobe connectivity and common genetic variants in *CNTNAP2* using a functional neuroimaging study^117^ and the study found that ASD and TD individuals who were nonrisk allele carriers showed more reduction in the activation of mPFC during an fMRI task as compared to risk allele carriers^117^. Another study showed decreased functional connectivity in the prefrontal cortex, cortical spinal tract, corpus callosum, and decreased integrity of WM in children and adolescents carrying *MET rs1858830, C* risk allele. Such studies suggest that the genes affect the brain regions that are involved in social and emotional processing^139^.

More detailed brain analysis combined with visualization, will promote the elucidation of the relationship between genes and altered neuronal circuits. Also, researching the time-dependent impacts of ASD-involved genetic variants that are the cause of neurodevelopmental modification can aid in the progress of ASD imaging genetics. In addition, the plasticity that is altered in different neuropsychiatric disorders such as ASD can be measured with high resolution by combining electroencephalography or fMRI with transcranial magnetic stimulation. Advances in noninvasive brain imaging techniques and scans during rest or task may help in infant neuroimaging that are at a higher risk to develop ASD. With studies investigating genetic effects on brain development using high-resolution imaging, studying brain-behavior connections and the impact of new therapies on brain development, further translational progress will happen in the future. By neuroimaging endophenotypes, a greater understanding of the impact of ASD risk genes on the brain circuitry^139^ can be achieved. Ultimately, imaging genetics in ASD might be promising for the clinical management of ASD-affected patients.

## Challenges and future perspectives

The field of imaging genetics has exponentially grown in recent decades from its candidate gene studies to large-scale longitudinal studies, cross-modal investigations, and translational animal models of various psychiatric disorders. In addition, imaging genetics has begun integrating transcriptomic data and analytical methods for assessing pathway enrichment, such as the score system for pathway regulation. Of addition to translational animal research and pharmacological intervention in vitro and in vivo, imaging genetics can also provide an insight into various behavioral and genetic factors that contribute to the risk of ASDs.

One of the challenges facing imaging genetics is the conceptualization of endophenotypes, which states that endophenotypes are heritable and associated with psychiatric disorders and may impede research on brain-based associations by limiting imaging genetic research to genes previously associated with a psychiatric disorder^140^. It is important to properly replicate the studies, particularly those with false-positive results, to address the impact of a genetic variation in a disease, and this problem can be solved by correcting genome-wide associations with large sample size imaging phenotypes.

Another challenge in ASD neuroimaging research is the discrepancy in the brain connectivity findings due to motion artifacts. The motion artifacts in the rsfMRI data can misrepresent group differences in connectivity metrics which can lead to incorrect interpretation of the altered brain connectivity data in ASD. Other methodological variables such as pipeline type, field of view, and dataset can significantly impact (in terms of over-or underconnectivity) the functional connectivity MRI studies in ASD^141^. Also, the differences in age and severity of the ASD cohort may lead to disparity in findings, and so large-scale studies are required that concentrate on exploring the developmental trajectories that are crucial to understanding the neurobiology underlying ASD. Such large-scale study databases can be obtained from various consortia, such as autism brain imaging data exchange and National Autism Research Database (NDAR). The heterogeneity of the ASD phenotypes, therefore, leads to the difficulties faced in ASD neuroimaging genetics as there is a high degree of variability in frequency, the number of behavioral symptoms reported, and the associated characteristics in ASD. In addition, the brain responses produced as a result of a particular task stimulus are inadequate to determine if these responses result from a neural processing disorder or are due to the introduction of an extrinsic factor.

In addition, more development in data analytical methods such as machine learning algorithms or software can allow data from multiple modalities to be incorporated into a heterogeneous population, and can also recognize and detect digital biomarkers^142^ that can be used for the clinical management of ASD patients.

## Conclusion

To explain the origin of ASD’s underlying pathophysiology, the study of genetic alterations and the relationship between genes and environmental factors is crucial. Imaging genetics studies have revealed important information about the pathophysiology of ASD. One of the key aims of imaging genetics studies in ASD is to elucidate neural pathways that give rise to phenotypical heterogeneity. Enhanced risk of ASD is shown to be related to several different single-nucleotide polymorphisms, and the stratification of neuroimaging data by specific genetic risk factors occurring in ASD may help to understand the neurobiology underlying this disorder. Such studies will help to elucidate ASD from various perspectives and subsequently will pave the way for personalized treatment for patients with ASD.
